# Advancing Parental Age and Risk of Solid Tumors in Children: A Case-Control Study in Peru

**DOI:** 10.1155/2018/3924635

**Published:** 2018-06-19

**Authors:** Ligia Rios, Liliana Vásquez, Mónica Oscanoa, Iván Maza, Jenny Gerónimo

**Affiliations:** Pediatric Oncology Unit, Edgardo Rebagliati Martins Hospital, 490 Domingo Cueto Avenue, Lima 11, Peru

## Abstract

**Background:**

The causes of childhood cancer are not well known, but the advanced age of the parents has been suggested as a risk factor for childhood cancer in several observational studies. In this study, we examine a possible link between parental age and childhood solid tumors.

**Methods:**

We conducted a hospital-based case-control study (310 cases and 620 controls, matched by age and gender) at Rebagliati Hospital, Lima, Peru. Odd ratio was used to compare categories of advancing maternal and paternal age with and without adjusting for possible confounding factors were calculated.

**Results:**

The risk of childhood retinoblastoma was significantly higher among children of mothers aged> 35 years (adjusted OR 1.21; 95% CI, 1.09-6.08) and fathers aged> 35 years (OR 1.17; 1.01-16.33). A significant trend with increasing mother's age (p = 0.037) and father's age (p = 0.005) was found. There were more risks to development of non-Hodgkin's lymphoma (p = 0.047) and gonadal germ cell tumors (p = 0.04) for advanced paternal age. There was a strong protective effect of increasing parity on risk of solid tumors in children (p=0.0015).

**Conclusion:**

Our results suggest that advanced parental age is associated with the risk for the development of retinoblastoma. Advanced paternal age increases the risk of non-Hodgkin lymphoma and gonadal germ cell tumor. The higher the order of birth of the children, the less the chance of developing any neoplasm.

## 1. Introduction

Although the childhood cancer is a rare disease, during the last years, appearance of children's cancer had a higher incidence rate, affecting approximately one in 435 children under age 15 years [1]. Several associated factors have been described in its development, like biological aspects (chromosomal anomalies, immunological alterations), environmental aspects (exposure to radiation, viral infections, socioeconomic status, and parental occupation), maternal aspects (breastfeeding, the mother's consumption levels of alcohol or tobacco, nutritional supplements [2–4]), and familial features (family history of cancer, advance maternal and/or paternal age, and number of previous siblings); however the etiology for the most of childhood cancer is still unknown.

Family structures have changed in relation to past generations since nowadays, when maternity/paternity occurs, advancing maternal or paternal age and a lower number of children are also more likely to occur. Additionally, advanced maternal age has been positively linked with higher risk of having a child with Down syndrome, among other congenital disorders, which in turn has a higher incidence of acute myeloid and lymphoid leukaemia [5]. Advanced paternal age (as a factor independent of maternal age) could mean risk of disorders associated germ cell mutation [6].

The link between advanced maternal and paternal age and a higher incidence of the appearance of children's cancer has been revealed in studies conducted among patients with leukaemia [7–9], lymphomas, brain tumors [10], germ cell tumors, and malignant neoplasms in general [11–13] even though, in many other studies, such link has not been corroborated with consistent results [14–18]. Likewise, some studies show an increase in the risk of childhood cancer in the first child [13, 14, 19] although, in many others, contradictory findings have been made [7, 20].

To date, it has not been made clear if advanced parental age is linked with higher risk of children's cancer; hence, the present study aims to determine whether advancing parental age is associated with an increased risk of childhood cancer in offspring.

## 2. Materials and Methodology

### 2.1. Participants and Recruitment

A case-control study based on hospital records (Rebagliati Hospital, Lima, Peru) was conducted. Both, cases and controls, included children and adolescents younger than 18. For every case, 2 age-and sex-comparable controls were assigned (in the case of the age-comparable control, the margin was +/- 6 months).

Cases had an anatomical pathological diagnosis of Hodgkin and Non-Hodgkin lymphoma, brain tumors, germ cell tumors, and any solid tumors, between the years 2012 and 2015. Controls were children who were hospitalized for nononcological diseases (ICD: A09, B01.9, G40.9, K35.9, J18.0, J21.9, J45.9, L03.9, S52, and T29) during the same period.

Data collection was conducted via questionnaires to parents about their medical histories and their families' medical histories, together with a revision of every patient or control patient's medical record.

### 2.2. Statistical Analysis

The data was analysed using conditional logistic regression for studies involving the cases and controls. Relative risks were estimated by odd ratios (OR) with a 95% confidence interval (CI) with a primary analysis between the link between maternal and paternal age as categorical variable according to age group (<20, 20-24, 25-29, 30-35, and >35) and as quantitative variable and childhood cancer diagnosis. We established raw OR and OR adjusted to confounding variables; these were chosen based on their prior observed association with childhood cancers [11–14]: paternal and maternal education (elementary, high school, and higher education), birth order of siblings in the family, and origin. Unfortunately due to missing data in control group, it was not possible to include birth weight and prematurity as confounding variables. Data was analysed with STATA statistical package (Small STATA Version 13.0, STATA Corporation, College Station, Texas, USA).

### 2.3. Ethical Aspects

This study has been evaluated and approved by the Ethics Commission of our institution prior to project execution. All clinical and sociodemographic data comply with confidentiality norms to protect the identity of patients.

## 3. Results

Between 2012 and 2015, 310 cases of childhood cancer (solid tumors and lymphomas) were identified; we compared those cases with 620 controls, matched according to age (with the margin of six months), sex, and geographical area of origin (Table 1).

The presence of childhood cancer was slightly higher among males. Parental age was grouped into a 5-year group. The main age of case mothers at birth was slightly older (28.92 [SD=6.30]) than control mothers (28.47[6.16]); contrary pattern was observed with the main age of fathers, slightly younger in cases (31.74 [6.82]) than controls (32.37 [7.82]). The only statistically significant difference between controls and cases was in the order of birth, since the difference observed in the level of education of the father is influence by the number of lost data.

Table 2 shows the distribution of solid tumors and lymphomas in the cases. Brain tumors (20%), non-Hodgkin lymphoma (13.87%), osteosarcoma (13.55%), and Wilms' tumor (11.29%) were the most frequent cancers. It should be mentioned that in our study Wilms tumor presented a higher prevalence than neuroblastoma (WT 11% versus NB 4%), unlike those occurring in North America and Europe, but similar to that observed in other studies in Latin America [21].

In our case-control study, maternal age shows positive linear trends (age groups are taken to be continuous) for 2 childhood cancer groups: osteosarcoma (p=0.019) and retinoblastoma (p=0.04); however after adjustment for covariates (paternal age, number of siblings, and level of parental education), maternal age only indicated a positive linear trend for retinoblastoma (p=0.037). In addition, the risk of development retinoblastoma was significantly higher among children of mothers older than 35 years old (adjusted OR: 1.21; 95% CI: 1.9-6.8); such association did not occur in other neoplasms. (Table 3).

Similarly, paternal age demonstrated positive linear trends (age groups are taken to be continuous) for 2 childhood cancer groups: gonadal germinal tumor (p=0.03) and retinoblastoma (p=0.03). After adjustment for covariates (maternal age, number of siblings, and level of parental education), paternal age continued showing a positive linear trend for non-Hodgkin lymphoma (p=0.047), gonadal germinal tumor (p=0.04), and retinoblastoma (p=0.037). Additionally, the risk of development retinoblastoma was significantly higher among children of fathers older than 35 years old (adjusted OR: 1.17; 95% CI: 1.0-16.33). (Table 4).

Furthermore, a protective effect against risk of solid tumors when parity increased (p=0.0015) was found. It was also observed that there was a significant decrease in the risk of children's neoplasms as the number of siblings in a family increased; there is a protective effect from the third child (OR adjusted: 0.63, 0.41-0.94). We decided to do the analysis of all the neoplasias together, due to having few cases to do a separated analysis. (Table 5).

## 4. Discussion

This work represents the first published study focused on advanced parental age (maternal or paternal) as a risk factor associated with the appearance of solid tumors in children in Peru.

Advanced parental age, considered in some studies as older than 35 or 40 years old, has been frequently linked with higher risk of children's malignant neoplasms [7–13], with results in other studies being contradictory [14–17]. Incidence rate of paediatric cancer is on the rise worldwide; likewise, advanced-age parity between mothers and fathers has become more common. The mechanism causing cancer in children of advanced-age mothers or fathers has not been clearly described. Reproductive age could affect risk of cancer in children through several processes, especially considering that as a parent becomes older, there are higher numbers of chromosomal mutations and aberrations in the maturation process of germ cells. In addition, changes in hormonal levels in the female reproductive system, as well as estrogenic levels, could bring about increased risk of children's neoplasms. It has been reported that paternal age is a factor for genetic problems, such as multiple endocrine neoplasia (MEN) and neurofibromatosis [22].

In our case-control study, we observed a slight rise in the risk of developing retinoblastoma by children of advanced-age mothers and fathers. For this cancer, the oldest maternal age group (>35 years) showed an elevated risk with adjusted OR de 1.21 (1.09-6-08) compared with other exposure age groups.

These results are similar to other studies. One of the most comprehensive cohort studies done by Yip et al. [12] describes that advanced maternal age, >40 years, is directly linked with higher risk of retinoblastoma in children (IRR= 2.42 1.04–5.58). Another population-based cohort study conducted by Moll et al. (1996) [23] observed that an increased risk of transmitting retinoblastoma was reported for mothers older than 35 (OR: 1.35, 95% CI: 1.06-1.72). Also, in a later population study, Foix-L'Hélias (2012) et al. [24] described that ageing mothers have an even higher risk of having children affected by retinoblastoma (OR: 2.42, 95% CI: 1.22-4.81).

However, the study of Johnson et al. (2009) [11] did not show increased risk among mothers over the age of 35 (OR: 0.93, 95% CI: 0.83-1.04). Like the study made by Dockerty et al. [13], that did not show higher risk for mothers between 35 and 39 years (OR: 1.08, 95% CI: 0.58-2.01) or mothers older than 40 years (OR: 1.30, 95% CI: 0.32-5.26). It is necessary to know that all these studies were population cohort, whereas our study had as controls a hospital population and had less population to study; these could cause some bias at moment of the analysis.

In the case of the effect of father's age, our study showed positive linear trend for the risk of retinoblastoma after adjusted by maternal age (p=0.005) and showed an increased risk in those parents who were older than 35 (OR=2.42, 95% CI: 1.04–5.58). These results were different to those found by Yip et al. [12] that did not have a relationship between advanced paternal age an retinoblastoma (p=0.165). In the same way, the study of Johnson et al. [11] concluded that the advanced paternal age is not a risk for development of retinoblastoma (OR: 1.01, 0.92-1.11). On the other hand, a study by Heck et al. (2012) [25] described that advanced father's age constituted a risk factor for the development of retinoblastoma (crude OR=1.73, 95% CI 1.20, and 2.47); they also mentioned that bilateral retinoblastoma risk was higher among children of older fathers.

In spite of those findings, general analysis of published studies does not make it clear that there is a fundamental difference in favour of advanced age of parents as the cause of increased risk of retinoblastoma among children.

In our study, apart from the positive association between advanced maternal age and risk of retinoblastoma, we did not find a relationship between advanced maternal and risk for other solid tumors, like as brain tumors, Wilms tumors, Ewing's sarcoma, soft tissue sarcoma, hepatoblastoma, Hodgkin lymphoma, and neuroblastoma. This is different to the report of Johnson et al. [11], who found positive linear trends for childhood cancer groups: lymphoma (1.06 per five-year increase [95% CI =1.01–1.12]), central nervous system tumors (1.07 [1.00–1.10]), neuroblastoma (1.09 [1.04 –1.15]), Wilms tumor (1.16 [1.09–1.22]), bone tumors (1.10 [1.00–1.20]), and soft tissue sarcomas (1.10 [1.04–1.17]). In models that adjusted to paternal age and other covariates, maternal age remained associated with childhood cancers overall, central nervous system tumors, neuroblastoma, Wilms tumor, and soft tissue sarcomas. Similar to our work, in other studies, there is no relationship between the parental age and risk of hepatoblastoma [11, 26, 27]. On the other hand, in our study we observed an increased risk of non-Hodgkin lymphoma and advanced paternal age (p = 0.047), unlike that described by Yip [12] et al., who did not find a significant association. In addition, we found a positive linear trend for the risk of gonadal germ tumor, after being adjusted by maternal age (p=0.005).

Interestingly, our study showed that single children had a higher risk of developing childhood cancer than children who had siblings. That is, the greater the order of birth of a child, the lower the risk of cancer (p=0.0015), and we could see a protective effect from the third child (OR adjusted: 0.63, 0.41-0.94), which continued for the fourth (OR: 0.58, 0.28-0.78) and fifth child (OR=0.20, 0.06-0.65). These results were similar to those reported by Von Behren et al. (2011) [21], who found that, for all combined cancers, there was a protective effect from the third child (OR=0.90, 0.85-0.96) and that it continued with the fourth child (OR=0.87, 0.81-0.93). Another study showed a rise in the risk of childhood cancer in the first-born child [19]; however, many other studies present contradictory findings [7, 12, 18, 20]. This information is crucial, since it could generate a main hypothesis in future studies.

Due to its observational nature, this study presents strengths and weaknesses. One of its main strengths is that it is the first study of its kind conducted in our country and region. Its main weakness lies in the number of cases included in the study, because these pathologies represent diseases, which are relatively infrequent. Further limitations also include employing hospital-type controls and missing data in some variables; therefore, the results obtained in this study cannot be generalized to the Peruvian population and are only useful in the hospital setting in which the study was carried out. Nowadays in Peru, it is virtually impossible to apply population studies into children's cancer because Peruvian hospitals do not have registration systems of optimal quality.

## 5. Conclusions

Our study suggests that advanced parental (maternal and paternal) age, ≥ 35 years old, increases the risk of developing retinoblastoma in children; at advanced paternal age increases the risk of developing non-Hodgkin lymphoma and gonadal germ cell tumors; and finally, this study observed that the higher the order of birth of the children, the less chance of developing any neoplasm. Additional studies with larger population-based samples are needed to confirm our hypothesis.

## Figures and Tables

**Table 1:** Characteristics of children diagnosed with cancer and controls (2012-2015).

**Characteristic**		**Cases**		**Controls**		**P value**
		**N**	%	**N**	%	

**Age, years**						0.119

	<1	16	5.2	42	6.8	

	01-04	83	26.8	145	23.4	

	05-09	76	24.5	167	26.9	

	10-14	87	28.1	171	27.6	

	15-18	48	15.5	95	15.3	

**Sex**						0.963

	Male	175	56.5	349	56.3	

	Female	135	43.6	271	43.7	

**Origin**						0.349

	Coast	233	75.2	485	78.2	

	Andean	59	19.0	95	15.3	

	Forest	18	5.8	40	6.5	

**Maternal age at birth, years**						0.239

	<20	17	5.5	36	5.8	

	20-24	88	28.4	138	22.3	

	25-29	72	23.2	194	31.3	

	30-34	81	26.1	135	21.8	

	>35	52	16.8	117	18.9	

	Mean (SD)	28.92 (6.30)		28.47 (6.16)		

	Missing	1		8		

**Paternal age at birth, years**						0.520

	<20	4	1.3	15	2.8	

	20-24	40	12.9	67	12.6	

	25-29	85	27.5	128	24.1	

	30-34	81	26.2	122	23.0	

	>35	99	32.0	199	37.5	

	Mean (SD)	31.74 (6.82)		32.37 (7.82)		

	Missing	1		89		

**Education level of the mother**						0.801

	Elementary school	26	8.5	22	9.6	

	High school	116	37.8	90	39.3	

	Higher education	165	53.8	117	51.1	

	Missing	3		391		

**Education level of the father**						0.039

	Elementary school	9	2.9	14	6.2	

	High school	100	32.7	87	38.5	

	Higher education	197	64.4	125	55.3	

	Missing	4		394		

**Birth order**						0.002

	1	188	60.8	354	57.1	

	2	84	27.2	175	28.2	

	3	26	8.4	63	10.2	

	4	10	3.2	15	2.4	

	>=5	1	0.3	13	2.1	

	Missing	1		0		

**Twins**						0.090

	Yes	2	0.6	7	1.1	

	No	308	99.4	613	98.9	

**TOTAL**		310	100.0	620	100.0	

**Table 2:** Distribution of solid tumors and lymphomas.

Characteristic		Cases	
		**N**	%

**Diagnosis**			

	Brain tumor	62	20.0

	Non-Hodgkin lymphoma	43	13.9

	Osteosarcoma	42	13.6

	Wilms tumor	35	11.3

	Ewing's sarcoma	24	7.7

	Soft tissue sarcoma	24	7.7

	Gonadal germinal tumor	21	6.8

	Retinoblastoma	16	5.2

	Hepatoblastoma	16	5.2

	Hodgkin lymphoma	14	4.5

	Neuroblastoma	12	3.9

	Extragonadal germinal tumor	1	0.3

**TOTAL**		**310**	**100.0**

**Table 3:** Effect of maternal age in childhood solid cancers development.

**Diagnosis**	**Maternal age**	**N**	**Unadjusted OR **	**CI 95**%	**Adjusted OR ** **∗**	**CI 95**%
**Brain tumor**	All ages	62	*p-value=0.99 * ***a***		*p-value=0.97*	

	<20	5	1.06	0.32-3.46	1.06	0.71-1.55

	20-24	16	2.06	0.80-5.29	1.92	0.27-4.16

	25-29***b***	14	1		1	

	30-34	18	2.10	0.83-5.27	1.42	0.53-3.08

	>35	9	1.26	0.44-3.55	0.88	0.26-2.97

**Non-Hodgkin lymphoma**	All ages	43	*p-value=0.56*		*p-value=0.80*	

	<20	1	0.33	0.03-3.08	0.45	0.34-4.50

	20-24	11	1.29	0.47-3.51	1.53	0.54-4.30

	25-29***b***	13	1		1	

	30-34	13	1.69	0.63-4.48	1.61	0.50-4.57

	>35	5	1.00	0.29-3-37	0.91	0.22-3.81

**Osteosarcoma**	All ages	42	*p-value=0.019*		*p-value=0.054*	

	<20	3	0.85	0.34-2.56	0.60	0.40-3.09

	20-24	16	0.70	0.24-2.04	0.58	0.16-2.08

	25-29***b***	10	1		1	

	30-34	8	1.38	0.52-3.64	0.99	0.62-2.30

	>35	5	1.91	1.12-3.09	1.35	0.81-2.33

**Wilms tumor**	All ages	35	*p-value=0.954*		*p-value=0.346*	

	<20	1	-		-	

	20-24	10	2.47	0.73-8.39	2.76	0.75-10.17

	25-29***b***	5	1		1	

	30-34	9	3.71	0.96-14.30	3.18	0.73-13.83

	>35	10	1.52	0.43-5.39	0.91	0.22-3.73

**Ewing's sarcoma**	All ages	24	*p-value=0.713*		*p-value=0.543*	

	<20	2	0.78	0.11-5.26	0.65	0.07-5.35

	20-24	7	1.24	0.32-4.76	1.51	0.31-7.37

	25-29***b***	7	1		1	

	30-34	3	0.35	0-07-1.60	2.73	0.51-1.33

	>35	5	1.46	0.26-8.11	1.05	0.18-6.06

**Soft tissue sarcoma**	All ages	24	*p-value=0.567*		*p-value=0.33*	

	<20	0				

	20-24	6	1.58	0.31-8.01	1.40	0.26-7.39

	25-29***b***	7	1		1	

	30-34	6	0.92	0.24-3.47	1.45	0.29-7.09

	>35	5	2.04	0.47-8.78	1.59	0.45-8.59

**Gonadal germinal tumor**	All ages	21	*p-value: 0.15*		*p-value=0.54*	

	<20	1				

	20-24	8	2.03	0.33-12.38	2.45	0.28-20.88

	25-29***b***	3	1		1	

	30-34	6	1.92	0.25-14.3	1.37	0.17-10.94

	>35	3	0.85	0.11-6.12	0.76	0.09-6.37

**Retinoblastoma**	All ages	16	*p-value=0.04*		***p-value=0.037***	

	<20	0				

	20-24	5	2.30	0.29-4.83	0.26	0.20-3.53

	25-29***b***	3	1		1	

	30-34	3	1.00	0.47-5.28	1.09	0.41-4.07

	>35	5	1.51	0.67-8.19	**1.21**	**1.09-6.08**

**Hepatoblastoma**	All ages	16	*p-value= 0.587*		*p-value=0.07*	

	<20	1	0.54	0.03-8.10	-	

	20-24	2	0.55	0.05-5.67	1.02	0.07-14-12

	25-29***b***	3	1		1	

	30-34	4	0.84	0.14-4.89	1.91	0.23-15.31

	>35	6	1.27	0.25-6.31	4.35	0.31-60.38

**Hodgkin lymphoma**	All ages	14	*p-value=0.192*		*p-value=0.196*	

	<20	2	5.08	0.34-75.08	5.58	0.20-14.93

	20-24	6	2.40	0.37-15.71	1.87	0.26-13.41

	25-29***b***	2	1		1	

	30-34	4	2.89	0.35-23.39	1.03	0.08-13.10

	>35	0				

**Neuroblastoma**	All ages	12	*p-value=0.898*		*p-value=0.51*	

	<20	1	-		-	

	20-24	1	-		-	

	25-29***b***	5	1		1	

	30-34	4	0.66	0.054-8-07	1.91	0.24-15.10

	>35	1	-		0.76	0.04-14.02

*∗ Adjusted by paternal age, number of siblings, and level of maternal and paternal education.*

*Significant results are bolded.*

***a*** *denotes where age groups are taken to be continuous in all cancers.*

***b*** *denotes reference group.*

**Table 4:** Effect of paternal age in childhood solid cancers development.

**Diagnosis**	**Paternal age**	**N**	**Unadjusted OR **	**CI 95**%	**Adjusted OR ** **∗**	**CI 95**%
**Brain tumor**	All ages	62	*p-value=0.94 * ***a***		*p-value=0.98*	

	<20	5	0.44	0.02-4.57	0.33	0.02-3.89

	20-24	16	1.12	0-42-3.01	1.01	0.36-2.83

	25-29***b***	14	1		1	

	30-34	18	0.90	0.33-2.4	0.91	0.30-2.73

	>35	9	0.90	0.36-2.19	0.99	0.34-2.85

**Non-Hodgkin lymphoma**	All ages	43	***p-value=0.09***		***p-value=0.047***	

	<20	1	-		-	

	20-24	11	0.24	0.06-0.96	0.19	0.03-0.97

	25-29***b***	13	1		1	

	30-34	13	0.54	0.17-1.73	0.37	0.09-1.43

	>35	5	0.48	0.17-1.34	0.36	0.82-1.64

**Osteosarcoma**	All ages	42	*p-value=0.10*		*p-value=0.23*	

	<20	3	-		-	

	20-24	16	5.70	0.98-33.0	3.10	0.94-8.6

	25-29***b***	10	1		1	

	30-34	8	1.80	0.55-5.24	1.15	0.32-4.05

	>35	5	1.35	0.46-3.97	0.92	0.27-3.1

**Wilms tumor**	All ages	35	*p-value=0.31*		*p-value=0.39*	

	<20	1	-		-	

	20-24	10	0.41	0.06-2.61	0.36	0.05-2.51

	25-29***b***	5	1		1	

	30-34	9	1.05	0.26-4.17	1.30	0.30-5.59

	>35	10	1.43	0.42-4.92	2.06	0.41-10.16

**Ewing's sarcoma**	All ages	24	*p-value=0.36*		*p-value=0.52*	

	<20	2	-		-	

	20-24	7	1.01	0.14-7.12	1.00	0.13-7.22

	25-29***b***	7	1		1	

	30-34	3	0.89	0.24-3.23	0.66	0.13-3.37

	>35	5	0.38	0.07-1.88	0.25	0.03-2.01

**Soft tissue sarcoma**	All ages	24	*p-value=0.43*		*p-value=0.38*	

	<20	0	-		-	

	20-24	6	0.33	0.28-4.11	0.91	0.4-18.3

	25-29***b***	7	1		1	

	30-34	6	0.48	0.11-2.02	0.76	0.13-4.3

	>35	5	0.42	0.12-1.45	0.25	0.02-1.86

**Gonadal germinal tumor**	All ages	21	***p-value=0.03***		***p-value=0.04***	

	<20	1	-		-	

	20-24	8	2.74	0-35-20.41	2.09	0.16-26.03

	25-29***b***	3	1		1	

	30-34	6	1.52	1.29-19.4	5.71	1.76-14.41

	>35	3	1.37	0.22-8.53	5.82	0.32-10.36

**Retinoblastoma**	All ages	16	***p-value=0-03***		***p-value=0.005***	

	<20	0				

	20-24	5	1.96	0.39-4.34	3.37	0.24-4.81

	25-29***b***	3	1		1	

	30-34	3	2.11	0.28-15.75	1.37	0.59-12.19

	>35	5	**2.10**	**1.21-15.04**	**1.17**	**1.01-16.33**

**Hepatoblastoma**	All ages	16	*p-value=0.16*		*p-value=0.21*	

	<20	1	-		-	

	20-24	2	-		-	

	25-29***b***	3	1		1	

	30-34	4	0.18	0.017-1.96	0.10	0.01-1.55

	>35	6	0.21	0.02-2.11	0.10	0.01-1.80

**Hodgkin lymphoma**	All ages	14	*p-value=0.97*		*p-value=0.47*	

	<20	2	0.72	0.03-16.01	0.24	0.01-11.58

	20-24	6	1.25	0.15-10.43	0.50	0.02-8.60

	25-29***b***	2	1		1	

	30-34	4	0.52	0.03-7.16	0.89	0.06-12.85

	>35	0	1.59	0.16-15.7	1.96	0.13-28.8

**Neuroblastoma**	All ages	12	*p-value=0.13*		*p-value=0.48*	

	<20	1	-		-	

	20-24	1	5.26	0.13-19.05	5.4	0.12-22.24

	25-29***b***	5	1		1	

	30-34	4	3.10	0.21-45.23	2.08	0.17-35.38

	>35	1	0.54	0.06-4.33	0.28	0.01-4.38

*∗ Adjusted by maternal age, number of siblings, and level of maternal and paternal education.*

*Significant results are bolded.*

***a*** *denotes where age groups are taken to be continuous in all cancers.*

***b*** *denotes reference group.*

**Table 5:** Effect of birth order in childhood solid cancers development.

Birth order	N	OR unadjusted	CI 95%	OR adjusted*∗*	CI 95%
**Trend **	310	***p-value=0.0010***		***p-value=0.0015***	

**1**		1		1	

**2**		0.88	0.64-1.23	0.87	0-62-1.21

**3**		**0.62**	**0.41-0.95**	**0.63**	**0.41-0.94**

**4**		**0.60**	**0.32-0.81**	**0.58**	**0.28-0.78**

**>=5**		**0.19**	**0.05-0.66**	**0.20**	**0.06-0.65**

*∗Adjusted by maternal age.*
